# Post-traumatic Stress Disorder in Resident Physicians

**DOI:** 10.7759/cureus.4816

**Published:** 2019-06-03

**Authors:** Theresa Lo, Lara De Stefano, Shaohua Lu, Vladimir Marquez-Azalgara, Kari-Jean McKenzie, George Ou, Eric Yoshida, Gary Lui

**Affiliations:** 1 Psychiatry, University of British Columbia, Vancouver, CAN; 2 Miscellaneous, St. George’s University, Grenada, USA; 3 Internal Medicine, University of British Columbia, Vancouver, CAN; 4 Gastroenterology, University of British Columbia, Vancouver, CAN; 5 Neurology, University of British Columbia, Vancouver, CAN

**Keywords:** ptsd, resident physicians, hospital, work place, mental health, anxiety, depression, mood disorders

## Abstract

Background

Research suggests that symptoms of post-traumatic stress disorder (PTSD) may be common in physicians who have experienced a traumatic event, but it is unclear if medical residents suffer from similar symptoms.

Objective

To determine the prevalence of PTSD symptoms in the resident physician population of the University of British Columbia based on the new Diagnostic and Statistical Manual of Mental Disorders-fifth edition (DSM-5) criteria.

Method

A link to an online questionnaire containing 27 questions, including residency training and year, as well as the PTSD Checklist for Diagnostic and Statistical Manual of Mental Disorders-fifth edition (PCL-5) was e-mailed and completed by the resident physicians of the University of British Columbia.

Results

Forty-three residents completed the survey and 38 had complete data. Mean PCL-5 score was 10.3 for the 38 subjects. Differences between PCL-5 score and resident year yielded the following: postgraduate year (PGY)-1=8.6; PGY-2=16.5; PGY-3=3.6; PGY-4=4.0; PGY-5=7.7. With respect to the type of traumatic event and PCL-5 score, the following was observed: Death=5.3, Violence=13.8, Medical Error=8.0, Bullying=38.0, None=45.0. The Kruskal-Wallis test showed no statistically significant differences in total PCL-5 score for PGY or type of traumatic event. Regardless of post-graduate year or trauma experience, four subjects out of 38 (10.5%) had a total PCL-5 score of 33 or greater, while one subject (2.5%) had a score greater than 50.

Conclusion

The results from this study conclude that resident physicians do suffer from PTSD symptoms at a rate higher than the average American population. As PTSD symptoms can often be very distressing and potentially affect work ethic negatively, further studies are indicated to better understand these symptoms and hopefully lead to better care in treating PTSD symptoms in resident physicians.

## Introduction

Post-traumatic stress disorder (PTSD) has been associated with multiple negative consequences including financial, occupational, social, marital, and health problems [1]. It is also a predictor for suicidal ideation and attempts [2-3].

According to the new criteria outlined in the Diagnostic and Statistical Manual of Mental Disorders-fifth edition (DSM-5), the definition of PTSD specifies that a traumatic event may involve repeated or extreme exposure to aversive details of the event [4].

Resident physicians, in their role as first responders, are witnesses to the consequence of violence, deaths, and serious injuries. During a typical five-year residency training program, most residents-in-training will be exposed to repeated averse medical events. There is no systematic evaluation of the risk of traumatic exposure to medical personnel, but it is well known that residents are at higher risk for stress, depression, and suicide when compared to the general population [5-6]. A study conducted on internal medicine residents in 2015 concluded that 28% characterized cardiopulmonary resuscitation (“codes”) events as traumatic, and 14% screened positive for at least one PTSD symptom such as intrusive thoughts, hyperarousability, irritability, avoidance or emotional numbing, all of which consequently affected work performance negatively [7].

A literature search using PubMed suggests that very little research has been conducted thus far regarding the prevalence of PTSD among medical professionals. One study of 212 American residents showed that 13% met diagnostic criteria for PTSD, which they attributed to stresses during their internship*. *This is much higher than the 8.7% projected lifetime risk for the general U.S. population [7-8]. As of yet, no studies of medical professionals have been conducted based on the new PTSD criteria as outlined in the DSM-5. There is also little data concerning Canadian residents and their risk for PTSD. The PTSD Checklist for Diagnostic and Statistical Manual of Mental Disorders-fifth edition (PCL-5) is a measure of PTSD symptoms using the DSM5 diagnostic criteria. Use of this measure will provide researchers with valuable information regarding the prevalence of residents suffering from PTSD symptoms using the most updated diagnostic criteria. (Abstract: Lo T, De Stefano L, Lu S, et al. Post-Traumatic Stress Disorder in Resident Physicians. Journal of Psychosomatic Research, June 2018).

## Materials and methods

We invited resident physicians undergoing training at the University of British Columbia to participate in an internet-based questionnaire between November 2016 and March 2017. Ethics approval was obtained from the University of British Columbia Behavioral Research Ethics Board. An email containing a link to the survey housed at the University of British Columbia’s Survey Tool webpage was sent through the faculty of Medicine’s head office with Professional Association of Residents and Interns of British Columbia (PARI-BC). From this secure website, participants were requested to anonymously complete the survey.

The questionnaire contained 27 questions: residency training and year (two questions); experiencing a traumatic event (five questions); and the PCL-5 (20 questions which correspond to the diagnostic symptoms outlined in the DSM-5). The PCL-5 is a self-reported survey that can be used to screen individuals and make a provisional diagnosis of PTSD. Significant research has been published on the PCL-5 with respect to validity, reliability, and consistency for each scale item [9-11]. For this study, a PCL-5 total score of 33 was used as a cut-point for the presence of possible PTSD symptoms, and a score of 50 or greater for probable PTSD symptoms. Statistical analysis was then conducted on the data to estimate the prevalence of PTSD amongst resident physicians by the level of training (postgraduate year (PGY)-1 through PGY-5) and by traumatic event (death, violence, medical error, and bullying). Qualitative responses were coded into categories based on consensus, while quantitative responses were examined for normality and missing data. Basic descriptive statistics were used to describe the data and nonparametric statistics were used to compare variables where possible. Data was analyzed using Statistical Package for the Social Sciences (SPSS), version 20 (IBM Corp., Armonk, New York, USA) with statistical significance set to p<0.05, two-tailed.

## Results

**Figure 1:**
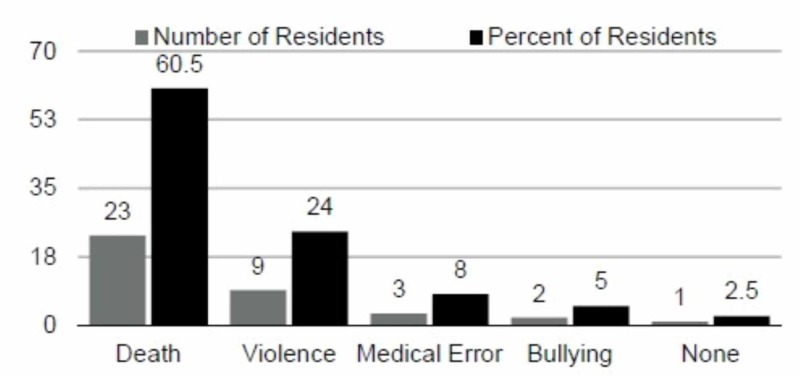
Type of Traumatic Event

A total of 43 residents completed the survey. The breakdown of seniority of residents was: PGY-1, 6; PGY-2, 17; PGY-3, 11, PGY-4, 3; PGY-5, 6; of these, 38 had complete data. Traumatic events were categorized as follows: Death (e.g., witnessing the death of a patient; consulting with family regarding a dying patient), Violence (being assaulted by a patient), Medical Error (witnessing a medical error), Bullying (experiencing harassment from a co-worker, or superior). Only one respondent (2.5%) indicated that he did not experience a traumatic event. The remaining participants (97.5%) were broken down as follows: Death (n=23; 60.5%), Violence (n=9; 24.0%), Medical Error (n=3; 8.0%), Bullying (n=2; 5.0%) (Figure 1).

**Figure 2:**
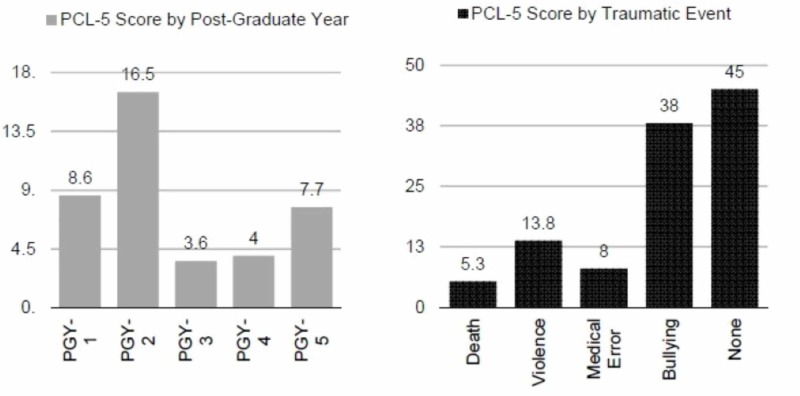
PCL-5 Score by Year and Traumatic Event PCL-5 = Post-traumatic stress disorder (PTSD) Checklist for Diagnostic and Statistical Manual of Mental Disorders-fifth edition

Mean PCL-5 score was 10.3 for the 38 subjects. Examining the differences between PCL-5 score and resident year yielded the following: PGY-1=8.6; PGY-2=16.5; PGY-3=3.6; PGY-4=4.0; PGY-5=7.7. With respect to the type of traumatic event and PCL-5 score, the following was observed: Death=5.3, Violence=13.8, Medical Error=8.0, Bullying=38.0, None=45.0. Kruskal-Wallis test showed no statistically significant differences in total PCL-5 score for PGY or type of traumatic event. Regardless of post-graduate year or trauma experience, four subjects out of 38 (10.5%) had a total PCL-5 score of 33 or greater, while one subject (2.5%) had a score greater than 50 (Figure 2).

## Discussion

The results from this survey suggest that PTSD symptoms may, in fact, be present in physician residents. The estimated projected lifetime risk for the general U.S. population to develop PTSD is 8.7% [8]. Based on the PCL-5 results of 38 resident physicians at the University of British Columbia, four subjects had a score of 33 or greater and one subject had a score greater than 50. This indicates that even in our small sample size, 10.5% (4 of 38 subjects) have the presence of possible PTSD symptoms and 2.5% (1 of 38 subjects) have probable PTSD symptoms, higher than the 8.7% risk the average American has. These results warrant further research into the evaluation of PTSD symptoms in medical resident physicians, and eventually a better support system for these residents. This study also concludes that supervisors should be vigilant of training residents as PTSD symptoms can be very distressing and can negatively impact the quality of life.

Our study has several limitations. The study was conducted via a completely voluntary online survey, thus possibly leading to a selection bias. It is unknown whether the individuals who completed the survey capture a real snapshot of the entire medical resident population, or whether the data is under-represented (the individuals who experienced a traumatic event were too busy or un-inclined to participate) or over-represented (the individuals who never experienced a traumatic event did not participate). There is also a possible limit to the generalizability of the study to the rest of Canada, as the sample only contained the University of British Columbia residents’ responses. Thus, findings from a single site with a relatively low response rate may further introduce a sampling bias. Lastly, the PCL-5 is only a screening tool, which although can provide a provisional PTSD diagnosis, is not definitive.

## Conclusions

The results from this study suggest that resident physicians do suffer from PTSD symptoms at a rate higher than the average American population. This suggests that further research may be needed in order to find the necessary tools to address this issue. It also suggests that supervisors should be more aware of resident trainees as PTSD symptoms can often be very distressing and potentially affect work ethic. Understanding PTSD symptoms in the medical workplace is extremely relevant and important as it has been shown that PTSD is associated with multiple health issues such as chronic pain and hypertension, and has a high comorbidity with other mental disorders.
